# Sublethal, sex-specific, osmotic, and metabolic impairments in embryonic and adult round stingrays from a location exposed to environmental contamination in southern California, USA

**DOI:** 10.1007/s11356-021-12546-0

**Published:** 2021-01-28

**Authors:** Kady Lyons, Katherine E. Wynne-Edwards

**Affiliations:** 1 Department of Biological Sciences, University of Calgary, 2500 University Dr. NW, Calgary, AB T2N 1N4 Canada; 2 Georgia Aquarium, 225 Baker St NW, Atlanta, GA 30313 USA; 3 Faculty of Veterinary Medicine, University of Calgary, 3280 Hospital Dr. NW, Calgary, AB T2N 4Z6 Canada

**Keywords:** Elasmobranch, Polychlorinated biphenyls, Pregnancy, Physiological effects

## Abstract

Organic contaminants are known to affect a suite of physiological processes across vertebrate clades. However, despite their ancient lineage and important roles in maintaining healthy ecosystems, elasmobranchs (sharks, skates, and rays) are understudied with regard to sublethal effects of contaminant exposure on metabolic processes. Perturbations resulting from contaminant exposure can divert energy away from maintaining physiological homeostasis, particularly during energetically challenging life stages, such as pregnancy and embryonic development. Using the round stingray (*Urobatis halleri*) as a model elasmobranch species, we captured adult males and pregnant females (matrotrophic histotrophy) and their embryos from two populations differing in their environmental exposure to organic contaminants (primarily polychlorinated biphenyls (PCBs)). Pregnant females from the PCB-exposed population experienced significant decreases from early- to late-pregnancy in tissue mass and quality not seen in reference females. PCB-exposed pregnant females also failed to maintain plasma urea concentrations as pregnancy progressed, which was accompanied by a loss in muscle protein content. Despite the energetic demands of late-term pregnancy, females had significantly greater liver lipid content than reproductively inactive adult males. PCB-exposed adult males also had high metabolic capacity (i.e., enzyme activity) for most substrate groupings of all sex-site groups, suggesting that males may be even more negatively impacted by contaminant exposure than pregnant females. Evidence that in utero exposure to PCBs via maternal offloading impairs embryo outcomes is accumulating. Embryos from the PCB-contaminated site had lower tissue quality measures and indications that sex-based differences were manifesting in utero as males had higher metabolic capacities than females. This study indicates that accumulated PCB contaminants are not physiologically inert in the stingray.

**Supplementary Information:**

The online version contains supplementary material available at 10.1007/s11356-021-12546-0.

## Introduction

Energetic fuels (i.e., carbohydrates, lipids, proteins) are needed to maintain physiological functioning not only for survival and reproduction but also for homeostasis for all living organisms. Environmental toxicant exposure can impose additional energetic costs on individuals (Beyers et al. 1999; Marchand et al. 2004; Smolders et al. 2005) that may result in the diversion of energy away from routine metabolism or other important physiological functions. Thus, any energetically demanding physiological process, such as growth, reproduction, immune function, or behavior, could potentially be impacted when organisms are exposed to toxicants (Adams et al. 1989; Little and Finger 1990; Feist et al. 2005). However, mechanistically linking toxicant exposure across various levels of biological organization (i.e., biochemical to whole organism) is challenging (Clements 2000; Adams et al. 2000), especially since not all species, sexes, or life stages will respond similarly to equivalent contaminant exposures (Elonen et al. 1998; Eisner et al. 2016; Li et al. 2019). Further challenges arise when contaminant effects are explored in non-model species, as physiological response to contaminant exposure may vary across taxa with different evolutionary histories. Toxicology studies on biochemical responses of contaminant exposure tend to focus on mammalian species, such as rats (Abdel-Daim et al. 2020; AlBasher et al. 2020) and mice (AlKahtane et al. 2020; Matelski et al. 2020), that serve as well-established models for understanding human health outcomes. However, a paucity of data exists for non-model species such as elasmobranch fishes (sharks, skates, and rays), despite the ecosystem services they provide as predators. Thus, examining effects of contaminant exposure in non-model species is valuable for holistic understanding of human impacts on ecosystem health.

Because physiological processes are naturally intertwined, disentangling the potential effects of contaminant exposure can be difficult, especially in wild populations where it is impossible to rigidly control situations to directly measure cause-effect relationships. Nevertheless, targeting specific biological situations with less energetic leeway may make it easier to detect effects of contaminant exposure in field-based studies. For example, pregnancy represents a period of substantial energetic investment (Gittleman and Thompson 1988) where females are more energetically taxed than in the non-reproductive parts of their life cycle. In elasmobranch fishes, females invest considerable energy resources to create large, well-developed offspring at the time of birth (Wourms and Demski 1993; Hamlett and Hysell 1998). For example, round stingray (*Urobatis halleri*) utilizes a form of matrotrophy (histotrophy), where the uterine lining secretes a nutrient-rich fluid that is consumed by young throughout development. Internal gestation with histotrophic matrotrophy represents a large energetic investment. In near-term round stingrays, litters account for 15–20% of their mother’s total mass (Lyons and Wynne-Edwards 2018). Thus, elasmobranch females would be especially sensitive during pregnancy to any additional costs imposed by other factors, such as energetic strain associated with toxicant exposure.

One major anthropogenic influence on the marine environment in southern California, a highly urbanized environment, is the existence of a United States Environmental Protection Agency Superfund site at the Palos Verdes Peninsula. Historic production and discharge have led to very high levels of dichlorodiphenyltrichloroethane (DDT) in the area as well as polychlorinated biphenyls (PCBs). While, sediment concentrations have declined after the cessation of chemical production in the early 1970s, since the 1990 concentrations have stabilized, which is attributed to bioturbation and the resuspension of contaminated sediment (Eganhouse and Pontolillo 2000; Eganhouse et al. 2000). These contaminants have accumulated to high levels in local biota (Allen et al. 2004; Blasius and Goodmanlowe 2008), resulting in recommended no fishing zones around the peninsula (Klasing et al. 2009) that are still in effect as of 2020^1^.

In southern California, a variety of adverse physiological effects have been identified in round stingrays exposed environmentally to PCBs but only minimally exposed to other anthropogenic environmental contaminants (Lyons et al. 2014; Sawyna et al. 2017). In these studies, stingrays were sampled from two wild populations where environmental PCB accumulation in hepatic tissues was significantly higher in mainland California (PCB-contaminated) animals than those from the offshore, genetically distinct (Plank et al. 2010) island population at Santa Catalina, despite the 4-year gap between each study’s collection period. Sex-related differences were identified in both the potential to accumulate contaminants (males had higher concentrations than females) and toxicity induction through the aryl hydrocarbon receptor, as males at the mainland site showed higher responses than their island counterparts but females showed no differences (Lyons et al. 2014). In addition, stingrays from the mainland population demonstrated elevated immunological endpoints compared to stingrays from the island, and PCB exposure was positively correlated with immunostimulation (Sawyna et al. 2017). With the implication of PCBs as the probable cause, these studies have established a study system between mainland California and a nearby offshore island for field-based studies to examine the outcomes of differing environmental contaminant exposure in elasmobranchs using the round stingray as a model.

Utilizing these paired populations, site and sex differences in physiological responses likely attributable to PCB exposure continue to be uncovered. Adult stingrays from the contaminated, mainland population have impaired primary and secondary stress responses relative to the reference, offshore island population (Lyons and Wynne-Edwards 2019b), with adult males more impacted than females, perhaps because they cannot offload toxicants to embryos. Furthermore, PCB exposure in utero is attributed to negative developmental growth impacts in male embryos but not females, suggesting sex-related differences of PCB effects may begin early in life (Lyons and Wynne-Edwards 2018). These two populations, therefore, have known biologically relevant impairments in the contaminant-exposed population. The physiological mechanisms behind those adverse impacts have not been studied.

As energy represents the currency of life, physiological processes that are costly may act as useful biomarkers, revealing how contaminants may metabolically disrupt biological systems. In adult fish, growth typically slows after maturity to accommodate the energetic costs of reproduction (Quince et al. 2008; Wilson et al. 2018) making size measures relatively insensitive markers of sublethal contaminant impacts. In contrast, osmoregulation is an expensive process across aquatic vertebrates (Bœuf and Payan 2001), with elasmobranchs utilizing a notably energy-demanding strategy to cope with the challenges of maintaining osmolarity against ambient seawater. Elasmobranchs produce and retain high levels of urea (Smith 1936; Evans et al. 2004). Urea has high energetic costs, both directly to produce (Ballantyne 1997) and to retain through specialized adaptations at the gills and kidneys to reduce loss (Smith and Wright 1999; Fines et al. 2001). Urea retention also has indirect costs for elasmobranchs since it has a destabilizing effect on protein tertiary structure and ultimately protein function (Rajagopalan et al. 1961). Elasmobranchs circumvent this challenge by maintaining elevated levels of trimethylamine-*N*-oxide (TMAO) relative to teleost fishes (Evans et al. 2004) in an optimal ratio of 2:1 urea to TMAO (Yancey and Somero 1980). Thus, because elasmobranch osmoregulatory processes are expensive, they are expected to be a sensitive physiological measure of contaminant impacts.

Contaminant impacts may also be detected during sensitive ontogenetic or life history windows (Vijayan et al. 2006), which may also reveal important metabolic processes that can be influenced by exposure (Zhang et al. 2012). For example, round stingray embryos from the PCB-exposed population exhibit slower growth and male, compared to female, embryos are relatively lighter than those of their reference counterparts (Lyons and Wynne-Edwards 2018). Given that embryonic development is a vulnerable period in an animal’s life (Elonen et al. 1998; Örn et al. 1998), contaminant exposure during pregnancy could impact embryos. Contaminants could have direct metabolic consequences for stingray embryos through maternal offloading (Lyons and Lowe, 2013) or indirect effects by straining female’s ability to provide proper nutrition to embryos during development.

In the current study, we hypothesize that PCB contamination will decrease liver energy reserves and increase hepatic metabolic capacity, and that urea-based osmoregulation will be compromised in response to legacy PCB contamination. Specific objectives are to assess biomarkers of (1) pregnant female liver metabolic capacity and tissue quality, (2) embryo metabolic capacity and tissue quality, and (3) urea-based osmoregulatory capacity. Since adult male stingrays carry higher levels of organic contaminants than females (Lyons et al. 2014), we also measure adult male tissue quality and metabolic capacity. This approach is expected to identify sublethal metabolic impacts of PCB exposure in a well-matched pair of wild elasmobranch populations differing in legacy PCB exposure and contaminant burden (Lyons et al. 2014; Sawyna et al. 2017; Lyons and Wynne-Edwards 2018, 2019b).

## Methods

### Study system

A benefit of utilizing southern California as a study system is the relatively close geographical juxtaposition of a contaminated site (mainland California) with a chain of offshore islands, providing a spectrum of anthropogenic influence on the local marine environment (Supplemental Fig. 1). Santa Catalina Island is located approximately 35 km from the southern California mainland. A deep ravine in the San Pedro Channel separates the island from the mainland, making it difficult for benthic-oriented species, without a pelagic phase as part of their development, or species with limited home ranges to cross from the mainland to the island. Current flow in the southern California Bight has contributed to limiting transport of contaminants from the Palos Verdes Shelf (EPA Superfund site) to the Island.

The current research used this two-site comparison between mainland southern California (PCB-exposed site; 33.731 N, 118.064 W) and Santa Catalina Island (reference site; 33.434 N, 118.503 W; Supplemental Fig. 1) as a natural study system to make potential inferences about PCB effects on elasmobranch physiology using the round stingray as a model. The geographic proximity of sampling locations, their equivalency to pure seawater salinity, and the availability of similar thermal refugia made it less likely these factors exerted a substantial influence on physiological differences between sites. While we did not quantify availability of prey (i.e., dietary factors) between the sites, muscle carbon and nitrogen stable isotopes in male stingrays from these locations showed no differences (Lyons et al. 2017), suggesting that stingrays from both localities likely feed on ecologically equivalent prey items. With genetic isolation between the two populations based on deep-water separation of sites (Plank et al. 2010), there was no expectation of cross-movement of individuals. The genetic markers used to determine separation between sites operate on drift, as microsatellites are chosen based on non-coding regions of DNA. Given the short time frame between the peak of PCB release into the environment and commencement of this study (44 years, ~ 10 generations of stingrays) as well as the relatively low rate of elasmobranch evolution relative to mammals (Martin et al. 1992), genetic divergence was not expected to play a major role in physiological responses to contaminant exposure between populations.

PCB exposure difference was previously established at these sites, and both DDT contamination and pharmaceutical contamination are minimal at the mainland site (Lyons et al. 2014, 2018; Sawyna et al. 2017). Since stingrays are isolated at the island in an environment that will have consistently less anthropogenic influence than the whole of southern California, we can be fairly confident that, despite an ability to move large distances, mainland stingrays will likely experience greater contaminant exposure for any given point in time than island stingrays. Thus, physiological differences between stingrays in this, and related studies, have been cautiously attributed to PCB exposure.

### Tissue sampling

All pregnant female (*n* = 59), embryo (*n* = 19 female, *n* = 21 male), and adult male (*n* = 18) samples were collected as part of a larger study examining the effect of environmental PCB exposure on stingray reproductive success (Lyons and Wynne-Edwards 2018, 2019a), and acute capture stress response (Lyons and Wynne-Edwards 2019b). Details of sampling procedures can be found in those works. Briefly, adult female stingrays were caught via hook and line every month of their reproductive cycle during pregnancy (June–September 2014) at the established PCB-exposed (mainland California) and reference (Santa Catalina Island) sites (Lyons et al. 2014; Sawyna et al. 2017; Lyons and Wynne-Edwards 2018). Once captured, females were subjected to one of two capture-handling treatments (representing a 15 min difference in time from hooking to euthanasia) to examine PCB impacts on the acute stress response (see Lyons and Wynne-Edwards 2019 or Supplemental Information of this article for more details). Prior to tissue sampling, stingrays were euthanized with an overdose of buffered tricaine methanesulfonate (MS-222) in accordance with approved protocols (University of Calgary Ethics Review #14-0016). Maternal disk width (size across the width body from wing tip to wing tip) was recorded, followed by whole blood (~ 5 mL), muscle (~ 2 g), and liver (whole) tissue sampling. Maternal dressed body mass (without internal viscera) and liver mass were recorded in the lab after returning from the field. Dressed mass was used to remove variability added from stomach or intestine fullness and variability contributed by other organ masses, leaving muscle as the major contributor to dressed mass. After maternal tissue sampling, a small slit was made in the left uterus and uterine fluid (histotroph) was aspirated (2–5 mL) and frozen. Embryos were then removed, individually wrapped in aluminum foil, and frozen for later dissection, where disk width and body, liver, and kidney mass were recorded. All tissues were frozen immediately after collection on dry ice in the field before being deep frozen (− 80 °C), except blood which was stored on wet ice until returning to the lab where plasma was separated and then frozen (− 80 °C).

Samples of muscle and liver tissue from PCB-exposed and reference males were obtained from concurrent (Lyons and Wynne-Edwards 2019b) and previous studies (Lyons et al. 2014) for comparison to late-term pregnant females (see “Data analysis”). Across studies, protocols for euthanasia and tissue preservation were similar such that, shortly after death, samples were extracted and immediately placed on dry ice before being stored at − 80 °C.

### Tissue extractions

Tissue homogenates were made from 96 ± 3 mg fresh weight of muscle and liver from each mother, one embryo from each litter developmentally advanced enough to allow liver sampling (> 3 g individual embryo total mass, clasper day 0; male-female sex ratio of sampled embryos: reference = 0.9:1, contaminated 1.3:1), and adult males. Muscle tissue (30% red, 70% white in adults, 100% white in embryos; K. Lyons pers. obser.) was excised from the ventral side of the left pectoral fin and ground to a fine powder by mortar and pestle on dry ice prior to adding it to a homogenization buffer (ice-cold solution 50 mM Tris buffer, pH 7.5) containing a protease inhibitor cocktail (Roche, Basel, Switzerland). Tissues in buffer were manually abraded then sonicated on ice for ~ 30 s and centrifuged at 5000 rpm for 2 min. The resulting lipid layer was removed and remaining supernatant was either split into aliquots and frozen (− 80 °C) for later use or added to a storage buffer (21 mM Na_2_HPO_4_, 0.5 mM EDTA, 0.2% bovine serum albumin, and 5 mM β-mercaptoethanol, 50% [v/v] glycerol, pH adjusted to 7.4; Vijayan et al., 1997) and stored at − 20 °C for future enzyme activity analysis.

### Tissue quality

For adults and embryos, protein and glycogen content were quantified in muscle and liver and lipid content was additionally measured in liver but not muscle as this tissue tends to be lipid poor in elasmobranchs (Lyons, unpublished data). Protein was quantified by the bicinchoninic acid method (Smith et al. 1985), glycogen as outlined by Bergmeyer (1983), and lipids via the Folch method (Folch et al. 1957). The energy content (kJ/g) for each liver fuel source was determined using coefficients from Jonsson et al. (1997) for proteins (17 kJ/g tissue), carbohydrates (17 kJ/g tissue), and lipids (38 kJ/g tissue). Total liver energy content was calculated by summing these fuel sources (kJ/g).

### Liver metabolic capacity

A battery of 8 enzymes representing key points in protein, lipid, and carbohydrate metabolic processing were quantified and grouped into categories based on the energy substrate on which they operate: proteins (aspartate aminotransferase; E.C. 2.6.1.1), alanine aminotransferase (E.C. 2.6.1.2), glutamate dehydrogenase (E.C. 1.4.1.2), carbohydrates (hexokinase; E.C. 2.7.1.1), lactate dehydrogenase (E.C. 1.1.1.27), phosphoenolpyruvate carboxykinase (PEPCK; E.C. 4.1.1.32), pyruvate kinase (E.C. 2.7.1.40), or ketone bodies (3-hydroxybutyrate dehydrogenase; E.C. 1.1.1.30).

Enzyme activities were measured in liver homogenates following previously described procedures (Mommsen and Walsh 1991; Wiseman and Vijayan 2011). Activities were directly calculated from the slope of the linear portion of the consumption of NADH produced or consumed (measured at 340 nm) on a plate reader (ThermoMax, Molecular Devices, Inc.) using accompanying SoftMax Pro 6 software and reported as μmoles of substrate consumed or product liberated per minute per g of liver protein (μmol/min/g). All assays were performed at room temperature (~ 23 °C). Previous works utilizing enzyme activities as a proxy for metabolism have traditionally compared enzymes individually among tissues or species (Dickson et al. 1993; Ballantyne 1997; Bernal et al. 2003). Since we were interested in overall metabolism, rather than activities of each specific enzyme, we used total liver metabolic capacity defined as the sum of all enzyme activities (μmol/min/g) within an individual as our metric to compare across groups. While we recognize the activity may not have an additive effect on metabolism, we use total metabolic capacity for simplicity as all enzymatic processes would in turn contribute to overall metabolism for each individual stingray. Individual enzyme activities are provided in Supplemental Information Table 1, Table 2, and Fig. 2.

### Osmoregulatory biomarkers

#### Urea synthetic capacity

With respect to enzymes of the urea cycle, glutamine synthetase represents the first step and arginase the last step. Glutamine synthetase and arginase activities were measured using frozen homogenate aliquots from embryo liver samples (*n* = 40) and a subset of female samples (*n* = 19). Activities were calculated colorimetrically as product produced over a specific interval of time against a standard curve (urea for arginase and glutamyl γ-hydroxamate for GSase; Mommsen and Walsh, 1991). Activity is reported as μmoles of product produced per minute per g of liver mass (wet weight).

#### Osmolytes

Urea was measured in each pair of pregnant female plasma and histotroph samples using methods outlined by Rahmatullah and Boyde (1980). Prior to analysis, each sample was deproteinated by adding trichloroacetic acid (TCA, 5% final concentration). A subsample of this solution was then diluted with MilliQ water and 3 mL of the chromogenic reagent was added to 100 μL of diluted sample. Samples were boiled in a water bath for 5 min and cooled, and the absorbance was measured at 525 nm with a urea standard curve (BioShop Canada, Inc.) run in parallel with samples using a 96-well plate reader (ThermoMax, Molecular Devices, Inc.). Similar procedures were followed for muscle homogenates in a subset of maternal muscle samples (*n* = 13), ranging from early- to late-pregnancy at both sites and in muscle tissue of one embryo starting from clasper day 0 (total mass ~ 3 g) from each site, where sufficient tissue could be extracted (reference *n* = 20; PCB-exposed *n* = 17). Urea was calculated on a per milligram tissue basis, corrected for the mass of muscle powder and volume of homogenization buffer.

TMAO was measured in plasma and histotroph samples as well as muscle homogenates specified above following the methods of Treberg et al. (2006), scaled down by one-quarter to conserve reagents. Samples were added to 9 volumes of ice-cold TCA and left on ice for 10 min before centrifugation (15.6*g* for 5 min) to separate precipitated proteins. The supernatant was removed and subsequently assayed by placing TCA extracts (300 μL) into a 2-mL tube followed by 300 μL toluene and 300 μL of the iron-EDTA mixture described by Wekell and Barnett (1991). Samples were capped, heated at 50 °C for 5 min, and cooled to room temperature before adding 600 μL of 45% KOH. Samples were vortexed for 15 s three times, allowing time in between mixing for separation of layers. The toluene phase was removed and added to a tube with sodium sulfate (~ 10 mg) before a solution of 0.02% picric acid in toluene was added. Samples were mixed and allowed to settle for 2 min before the samples were read at 410 nm on a SmartSpec Plus spectrophotometer (Bio-Rad) using a quartz cuvette. A standard curve (TMAO•2H_2_O; Sigma Aldrich) was also run in parallel to samples. Osmolality of plasma and histotroph samples was measured using a Precision Systems Inc. Osmometer (model number 5004).

### Data analysis

Energetic demands on pregnant females were not anticipated to be linear as embryo reliance on maternally derived supplemental nutrition sources undergoes specific shifts across pregnancy (Hamlett and Hysell 1998). During early-pregnancy, embryos are mostly reliant on yolk-derived nutrient resources (i.e., energy that females have already expended) with little need for additional resources. Thus, additional energetic demands on females should be low during this stage. However, as embryos quickly deplete yolk reserves, they transition from partial reliance (mid-term) on supplemental nutrition (i.e., histotroph) to full reliance (late-term). The last stage of development was anticipated to be the most energetically taxing not only because females must create a new resource (histotroph) but also at a rate supporting the exponential growth of embryos during this period. Therefore, female parameters were compared between sites by pregnancy stage (early, mid, late) based on embryo development described above using two-way ANOVAs and post hoc testing with Bonferroni correction so that analyses would occur a biologically relevant context of maternal energetic demands. Pearson correlations were used to identify linear associations within stages, when appropriate.

Unlike the energetic demands placed on mothers that we predicted to be stage-specific, embryos are continuously undergoing developmental changes over the course of gestation (Babel 1967). Therefore, we wanted to capture developmental change between sites through a temporal variable. However, the reproductive asynchrony between populations (females at the contaminated site ovulated earlier than reference females) necessitated an alignment metric. To correct for differences in development initiation between sites, clasper days (days relative to external male copulatory organ appearance) were used to developmentally align litters (Lyons and Wynne-Edwards 2018). Tissue quality parameters, metabolic capacity, and osmoregulatory biomarkers were then compared along this continuous variable for embryos through ANCOVAs with clasper day, sex, and site as independent factors. When clasper day (i.e., development) was not a significant factor, parameters were compared through two-way ANOVAs or Welch’s *t* tests, depending on the number of significant factors.

Prior to pregnant female and embryo analyses, the potential effect of our imposed capture stressor was determined. Except for the expected depletion of free glucose in response to acute capture stress occurring in the last 15 min before sampling (Lyons and Wynne-Edwards 2019b), the imposed capture stress did not affect the parameters quantified in this study (all *p* ≥ 0.07). Thus, stress was not included as a factor in our analyses.

To investigate sex-related differences in adult stingrays, we compared parameters in testicular-quiescent (not reproductively active) males and late-term females, which would presumably correspond to height of energetic-demand disparity between males and females. PCB-exposed and reference male samples from the months of July and August were obtained from a concurrent study (Lyons and Wynne-Edwards 2019a) and the reference male group was supplemented with tissues collected previously (Lyons et al. 2014) to achieve comparable sample sizes. Individual parameters of adult tissue quality and metabolism were compared using two-way ANOVAs and post hoc testing with Bonferroni correction. Pearson’s correlations were used to examine the association of tissue quality parameters with metabolic capacity based on substrate groupings.

All analyses were performed using R statistical package (V 3.4.1) (R Core Team 2020) with *α* set to 0.05. For all tests, data were checked to meet assumptions of normality and homogeneity and non-conforming parameters were natural-log transformed as needed.

## Results

As described previously (see Lyons and Wynne-Edwards 2018), liver tissue from a subset of females from the PCB-exposed population had significantly higher levels of PCB contaminants compared to that from a subset of reference females (Welch’s *t* test, *p* = 0.015, 2856 ± 3353 versus 817 ± 1019 ng/g lw), confirming previous, independent results (Lyons et al. 2014; Sawyna et al. 2017). Among PCB contaminants detected, dioxin-like (i.e., planar) PCBs contributed little to overall PCB concentrations for PCB-exposed (1–24.3%, median = 3.3%) and reference females (0–10.3%, median = 0%). Furthermore, no response was found between summed concentrations of dioxin-like PCBs and relative CYP1A expression (Supplemental Fig. 3A–C). Of the 53 PCB congeners and 30 pesticides analyzed, PCBs contributed overwhelmingly to total contaminant concentrations regardless of site (exposed: 84 ± 5.7%, reference: 86 ± 8.8%). Thus, of the contaminants that were screened, PCBs were established as the major toxicant distinguishing the two sites, which otherwise shared geographical proximity and tidal flushing with full seawater. While adult males were not analyzed in this study, previous reports have demonstrated significant differences in contaminant accumulation similar to females (Lyons et al. 2014; Sawyna et al. 2017).

### Mothers

**Fig. 1:**
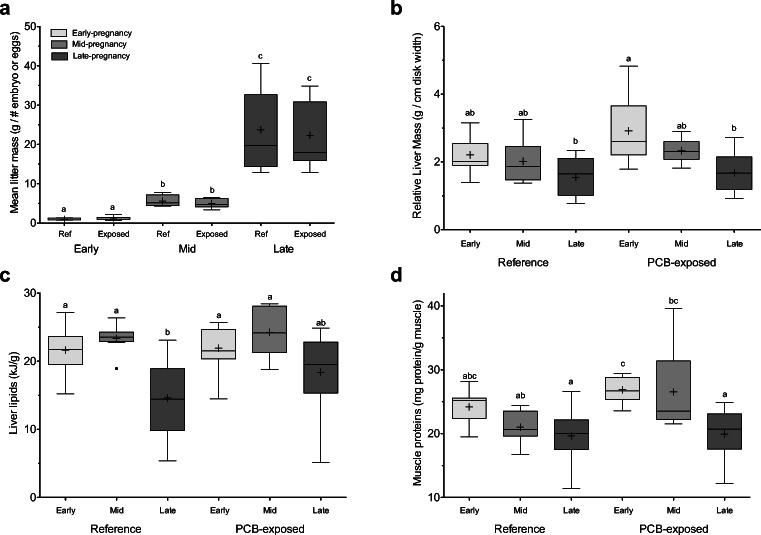
**a** Energetic demands placed on mothers are reflected in mean litter mass changes across pregnancy stages (early-term: light gray, mid-term: medium gray, late-term: dark gray). Pregnancy and site had corresponding effects on maternal **b** relative liver mass, **c** liver lipid content, and **d** muscle protein content across pregnancy stages and sites. Significant differences from two-way ANOVAs and Bonferroni post hoc correction for each parameter across groups are denoted with different letters. Whiskers of boxplots represent 1.5 times the interquartile difference and crosses within group means

Pregnant females from the reference (*n* = 29) and PCB-exposed (*n* = 30) sites were sampled from June (post-ovulation/early-pregnancy) through September (late-pregnancy) (Lyons and Wynne-Edwards 2018). While mean litter mass increased each stage of pregnancy as expected, the degree of increase was not equal between stages. From early- to mid-pregnancy, mean litter mass increased by ~ 95% for both sites; however, from mid- to late-pregnancy, mean litter mass increased by over 300% (Fig. 1a), highlighting the increased resource demand placed on mothers.

#### Tissue quality

Both tissue quantity (i.e., mass) and tissue quality (i.e., composition) decreased with the advancement of pregnancy. For relative liver mass, pregnancy stage (two-way ANOVA, *p* < 0.0001) and site (*p* = 0.038) had significant effects. In PCB-exposed females, mean relative liver mass (g liver/cm disk width) decreased from early- to late-term (Fig. 1b), while reference females experienced no significant changes between the beginning and end of pregnancy. Unlike the liver, relative body mass (g dressed mass/cm disk width) did not change over pregnancy at either site (two-way ANOVA, *p* > 0.4). The significant changes in relative liver mass, but not body mass, suggest that the liver bears the brunt of pregnancy and is a sensitive indicator of maternal condition. This was not due to simple starvation, as all maternal stomachs showed recent feeding. However, no information was collected about prey species and quality.

Because liver lipids contributed the most to total liver energy (92 ± 4.7%), the significant effect of pregnancy stage was similar for these two components (two-way ANOVA, *p* ≤ 0.0001), with no effect of site (*p* ≥ 0.2). While mean decreases in liver lipids (and total energy) occurred during the late stage at both sites (Fig. 1c), post hoc testing revealed late-term reference females to have significantly lower lipid content (and total energy) than early- or mid-term reference females (*p* = 0.047 and *p* = 0.005, respectively). Within late-stage females, we explored associations between liver lipid content and measures of maternal resource investment (i.e., total litter biomass, mean litter mass). For PCB-exposed females only, liver lipid content decreased as total litter biomass increased (*t*_11_ = − 2.4, *p* = 0.034, *r* = − 0.59), although no association was found with mean litter mass (*p* = 0.066).

While liver represents the main storage organ for lipid, multiple protein caches are present throughout the body that females may draw to provide supplemental nutrition to embryos. Liver protein content remained unaffected by pregnancy stage or site (two-way ANOVA, *p* ≥ 0.1), whereas muscle protein was significantly affected by both main factors (*p* < 0.0001 and *p* < 0.021, respectively) (Fig. 1d). Reference females did not experience a change in muscle protein from early- to late-term (*p* > 0.1); however, levels declined in PCB-exposed females from early- to late-term (*p* < 0.001). Transient mediums, such as plasma and histotroph, showed incongruent patterns. For plasma, pregnancy stage and site had no effect on protein content (*p* ≥ 0.3); however, among late-term reference females, there was a negative association between mean litter mass and plasma protein content (*t*_8_ = − 2.4, *p* = 0.039, *r* = − 0.66). Histotroph protein content, on the other hand, experienced significant effects of stage (two-way ANOVA, *p* < 0.0001) and a weak effect of site (*p* = 0.045), with protein content dropping significantly from early- to mid-term at both sites. No correlations were found between muscle and histotroph or plasma protein content within each stage of pregnancy for either site (*p* ≥ 0.1).

Glycogen stores in both liver and muscle were significantly affected by pregnancy stage (two-way ANOVA, *p* = 0.011 and *p* = 0.0001, respectively) with an additional weak effect of site for muscle stores (*p* = 0.05). While mean liver glycogen content appeared to decrease with stage, post hoc testing demonstrated no significant differences among pregnancy stages or sites (*p* ≥ 0.15). Muscle glycogen exhibited stronger responses to pregnancy stage in PCB-exposed females as lowest levels were found in late-term females compared to early- and mid-term (*p* ≤ 0.018). In reference females, muscle glycogen did not drop significantly as pregnancy progressed (*p* ≥ 0.34).

#### Liver metabolic capacity

Liver total metabolic capacity was not affected by pregnancy or site (*p* ≥ 0.3); however, late-term PCB-exposed females were the only group to experience a negative correlation between liver total energy and total metabolic capacity (*t*_13_ = − 2.4, *p* = 0.029, *r* = − 0.56). Among substrate groups, site but not pregnancy stage was a significant factor influencing carbohydrate and ketone metabolic capacity (two-way ANOVAs, *p* ≤ 0.008). For both substrate groups, PCB-exposed females had higher enzyme activities than reference females for both carbohydrate (Student’s *t* test, *t*_57_ = − 3.2, *p* = 0.002) and ketone (*t*_57_ = − 3.6, *p* = 0.0006) metabolic capacities. Protein metabolic capacity was unaffected by site or pregnancy stage (*p* ≥ 0.2); however, protein metabolic capacity was negatively correlated with liver protein content at each stage of pregnancy for PCB-exposed females (*p* ≤ 0.0001), while these same parameters were only negatively correlated during early-pregnancy in reference females (*p* < 0.012; Supplemental Fig. 4A–C).

#### Osmoregulatory biomarkers

**Fig. 2:**
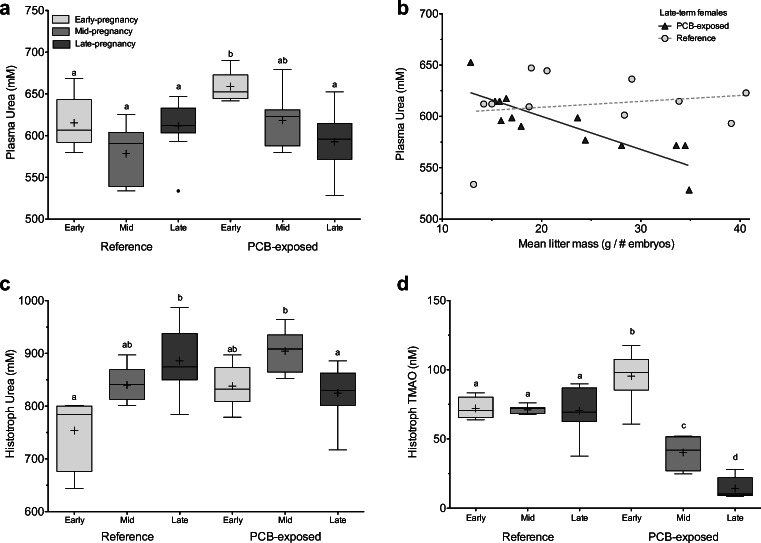
Urea, the main osmolyte utilized by elasmobranchs, was compared in maternal tissues for **a** plasma and **c** histotroph for reference (circles) and PCB-exposed (triangles) females by pregnancy stage (early-term: light gray, mid-term: medium gray, late-term: dark gray). **b** In late-term, PCB-exposed pregnant females (triangles, solid line), a significant negative correlation existed between mean litter mass and plasma urea compared to late-term reference females (circles, dashed line). **d** Changes in histotroph TMAO across pregnancy are shown for reference and PCB-exposed samples. Significant differences from two-way ANOVAs and Bonferroni post hoc correction for each parameter across groups are denoted with different letters. Whiskers of boxplots represent 1.5 times the interquartile difference and crosses within group means

Plasma from pregnant females was obtained in equal numbers at both sites (*n* = 28); however, due to six spontaneous uterine expulsions after euthanasia but before histotroph sampling, histotroph was only collected from 26 PCB-exposed and 24 reference females. Plasma urea was significantly affected by pregnancy stage (two-way ANOVA, *p* = 0.008) with a weak effect of site (*p* = 0.052) that led to a significant interaction (*p* = 0.001). As pregnancy progressed, plasma urea fell significantly from early- to late-term in PCB-exposed females (*p* < 0.0001), but not reference females (*p* > 0.7; Fig. 2a). Within late-term, PCB-exposed females, plasma urea had negative associations with both mean litter mass (*t*_11_ = − 5.5, *p* = 0.0002, *r* = − 0.86) and total litter biomass (*t*_11_ = − 3.2, *p* = 0.009, *r* = − 0.69; Fig. 2b), correlations which were not significant during early- or mid-pregnancy highlighting the strain placed on late-term females. Despite the significant changes in plasma urea, plasma osmolality, TMAO, and urea:TMAO ratios were unaffected by pregnancy stage or site (*p* ≥ 0.13).

Unlike plasma, the uterine environment also showed significant changes across pregnancy for both urea and TMAO and their ratio. Pregnancy stage and its interaction with site had significant effects on histotroph urea concentrations (two-way ANOVA, *p* = 0.01 and *p* = 0.0001). In reference females, urea histotroph increased from early- to late-pregnancy (*p* = 0.0006), likely as a result of nitrogenous-waste excretion by growing embryos. In contrast, histotroph urea concentrations in PCB-exposed females fell from mid- to late-pregnancy (*p* = 0.038; Fig. 2c). In addition, histotroph TMAO was significantly affected by both pregnancy stage and site and their interaction (two-way ANOVA, *p* ≤ 0.0001). For PCB-exposed females, mean histotroph TMAO experienced step-wise decreases across pregnancy (*p* ≤ 0.01), whereas reference females experienced no change in concentrations (*p* ≥ 1.0; Fig. 2d). Correspondingly, urea:TMAO ratios were also significantly affected by pregnancy stage, site, and their interaction (two-way ANOVA, *p* ≤ 0.0001). By mid-pregnancy, histotroph from PCB-exposed females had significantly higher urea:TMAO ratios over reference samples (*p* ≤ 0.011), while reference samples experienced little change over pregnancy (*p* = 1.0). Surprisingly, histotroph osmolality was not affected by pregnancy, site, or their interaction (*p* ≥ 0.2) suggesting that other unmeasured osmolytes may have had a compensatory effect on histotroph osmolality in the face of urea and TMAO changes.

#### Other potential osmoregulatory challenges

A subset of PCB-exposed and reference female samples was analyzed for muscle urea and TMAO (*n* = 12) and liver urea cycle enzymes (*n* = 19). While our subset captured a limited amount of information, we found indications that PCB-exposed females may have an impaired ability to maintain muscle urea concentrations compared to reference females across pregnancy (Supplemental Fig. 5); however, further study is needed to confirm this pattern. In addition, urea production capability may also be limited in PCB-exposed females compared to their reference counterparts. Enzyme activities for two important steps in the urea cycle were marginally (glutamine synthetase, *p* = 0.08) and significantly higher (arginase *t*_17_ = 2.4, *p* = 0.026) in reference females compared to those in PCB-exposed females. No correlations were found between plasma urea and arginase activity in reference females (*p* = 0.22), while PCB-exposed females exhibited weak negative correlations between arginase activity and plasma urea (*p* = 0.065 *r* = − 0.64). Increased sampling across pregnancy would be needed to determine with more confidence the association between urea production capability and contaminant exposure.

### Embryos

#### Tissue quality

**Fig. 3:**
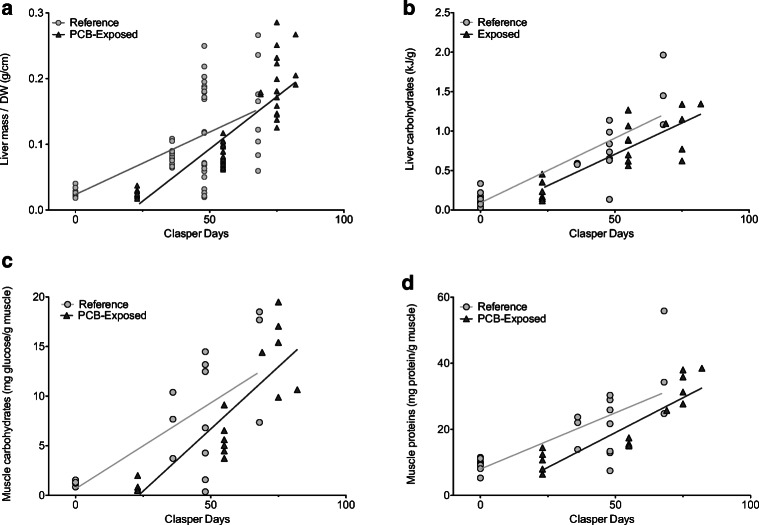
Metrics of embryo quality for **a** relative liver mass across all embryos from all litters and one embryo from each litter for **b** liver carbohydrate energy content, **c** muscle carbohydrate content, and **d** muscle protein content. Reference litters are shown as gray circles and PCB-exposed embryos as dark gray triangles. Significant relationships are denoted with solid lines. Site was a significant factor for analyses in each panel such that y-intercepts differed between groups (slopes were similar)

Starting at clasper day 0 (total mass ~ 3 g), livers were excised from one embryo from each litter associated with reference (*n* = 21) or PCB-exposed (*n* = 19) pregnant females. Since relative embryo liver mass (g/disk width) data were available for all embryos in a litter, all embryos were included to test for the effect of development and site on this metric. We accounted for the non-independence of multiple embryos per litter using a nested random effect of embryos/mother in our linear mixed model (*nlme* R package). Development (*p* < 0.0001) and site (*p* = 0.017) had significant main effects with no interaction (*p* = 0.12), with PCB-exposed embryos having slighter livers for their developmental stage compared to reference embryos (Fig. 3a).

Although total liver energy content (kJ/g) increased across development (ANCOVA, *p* = 0.004), with no effect of site (*p* = 0.96) or their interaction (*p* = 0.15), individual substrate groups showed differences. Although liver carbohydrate content (kJ/g) increased across development (ANCOVA, *p* < 0.0001), site had a significant effect (*p* = 0.032) with no interactions (*p* = 0.9) such that values were significantly lower in PCB-exposed embryos than in reference embryos (i.e., different y-intercepts; Fig. 3b). In contrast, development was the only significant factor on liver lipid content (kJ/g, ANCOVA, *p* = 0.019) and liver protein content (kJ/g, ANCOVA, *p* = 0.0002), with no effect of site (*p* ≥ 0.59) and no interaction (*p* ≥ 0.084).

Similar to liver, although more pronounced, embryo muscle quality also showed an effect of development and site. Muscle carbohydrates (mg glucose/g muscle) significantly increased with development (ANCOVA, LN-transformed, *p* < 0.0001) and had a weak effect of site (*p* = 0.043), with no interaction (*p* = 0.11; Fig. 3c). Muscle protein content (mg protein/g muscle) also increased with development (ANCOVA, LN-transformed, *p* < 0.0001), with an effect of site (*p* = 0.021) and no interaction (*p* = 0.39; Fig. 3d). For both parameters, PCB-exposed embryo muscle tissue quality was lower than of reference embryos (i.e., lower intercepts).

#### Liver metabolic capacity

**Fig. 4:**
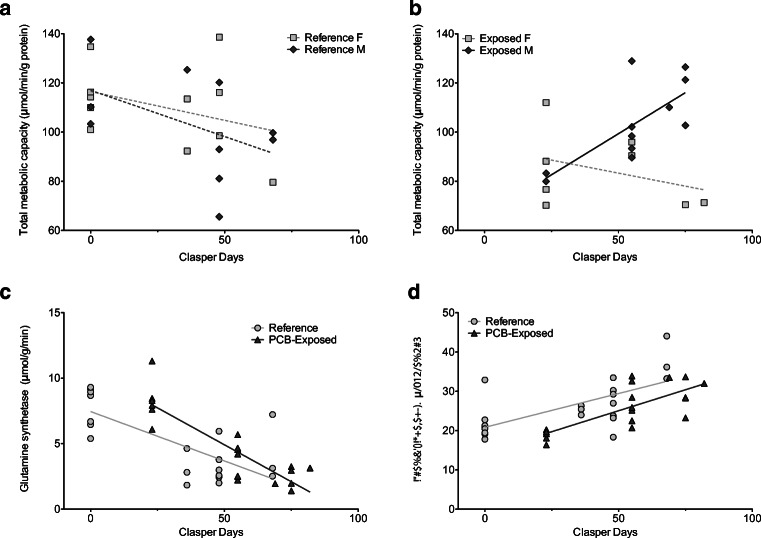
Since total metabolic capacity had a significant interaction with development, sex, and site, data is shown separately for embryonic males (diamonds) and females (squares) from the reference (**a**) and PCB-exposed (**b**) sites. Activity of enzymes related to the urea cycle is shown for the first step, glutamine synthetase (**c**), and the last step, arginase (**d**), for embryos from the reference (circles) and PCB-exposed (triangles) sites across development. Significant relationships are denoted with solid lines and trends (i.e., insignificant relationships) in dashed lines. For panels **c** and **d**, only site had a significant effect as y-intercepts were different between groups (slopes were similar)

Unlike tissue quality, total metabolic capacity had no main effects of development (ANCOVA, *p* = 0.26) or embryo sex (*p* = 0.28), but a weak main effect of site (*p* = 0.076), which lead to significant interactions (*p* ≤ 0.042). For reference embryos, males and females showed weak decreases in total metabolic capacity with development (ANCOVA, *p* = 0.074; Fig. 4a), and no effect of sex (*p* = 0.63) or their interaction (*p* = 0.66). PCB-exposed embryos also showed no effect of development (ANCOVA, *p* = 0.27); however, there was a significant effect of embryo sex (*p* = 0.018) and an interaction (*p* = 0.011). In PCB-exposed male embryos, total metabolic capacity increased with development (*F*_1,9_ = 10.73, *p* = 0.009, *r*^2^ = 0.49), while PCB-exposed female embryos did not show any effects of development (*p* = 0.38; Fig. 4b). In addition, PCB-exposed male embryos were also the only sex-site group to exhibit a significant, positive correlation between total metabolic capacity and total hepatic energy content (*p* = 0.003, *r* = 0.08; all others *p* ≥ 0.09).

Among substrate groups, protein metabolic capacity was the only group to show significant effects of development (ANCOVA, *p* = 0.034), without main effects of sex (*p* = 0.29) or site (*p* = 0.11), and a significant interaction only between development and site (*p* = 0.022). As reference embryos grew, liver protein metabolic capacity significantly decreased (*F*_1,19_ = 11, *p* = 0.004, *r*^2^ = 0.33), while PCB-exposed embryos exhibited no changes in liver protein metabolic activities across development (*p* = 0.39). Ketone metabolic capacity was not affected by site, sex, or their interaction (ANCOVA, *p* ≥ 0.13), and only weakly increased with development (*p* = 0.055). Carbohydrate metabolic capacity was the only substrate group to show no main effects of development, site, or sex (*p* ≥ 0.11) or their interactions (*p* ≥ 0.079).

#### Osmoregulatory biomarkers

Unlike their mothers, embryo muscle urea concentrations remained unaffected by development or site (ANCOVA, *p* ≥ 0.37; Supplemental Fig. 6A). In contrast, muscle TMAO significantly increased with development (ANCOVA, *p* = 0.004) and had an effect of site (*p* = 0.02) with no interaction (*p* = 0.9), as PCB-exposed embryos had significantly lower concentrations than reference embryos (i.e., lower y-intercept; Supplemental Fig. 6B). Nevertheless, muscle urea:TMAO ratios significantly decreased across development (ANCOVA, *p* = 0.0002) with no effect of site (*p* = 0.09) or their no interaction (*p* = 0.44). Although embryo muscle urea:TMAO ratios were much higher than adults during early development (4.05 ± 0.55 versus 1.93 ± 0.34), they reached ratios closer to adult values and the desired 2:1 ratio (Yancey and Somero 1980) towards the end of development (all late-development embryos: 2.77 ± 0.37; Supplemental Fig. 6C). Embryo muscle urea and TMAO concentrations were not correlated with histotroph concentrations for reference (*p* = 0.7 and *p* = 0.2) or PCB-exposed samples (*p* = 0.5 and *p* = 0.08), suggesting that embryos may have been able to self-regulate internal osmolyte levels.

#### Urea metabolism

Site and development had significant effects on urea cycle enzymes in utero. Glutamine synthetase activity declined across development (ANCOVA, *p* < 0.0001) with a significant effect of site (*p* = 0.016) and no interaction (*p* = 0.12). PCB-exposed embryos had higher activities of this enzyme than their reference counterparts (i.e., elevated y-intercept; Fig. 4c). In contrast, arginase activity, the last urea-producing step, significantly increased with development (ANCOVA, *p* < 0.0001), and also had a significant effect of site (*p* = 0.006) and no interaction (*p* = 0.52), with PCB-exposed embryos having lower arginase activities than reference embryos (i.e., lower y-intercept; Fig. 4d). Arginase activity had a weakly positive association with histotroph urea in reference samples (*p* = 0.084, *r* = 0.4), but no association in PCB-exposed samples (*p* = 0.13). The differences in urea production suggest PCB-exposed embryos may have lower capabilities to produce this important osmolyte compared to their reference counterparts prior to birth.

#### Effect of sex into adulthood

In adult stingrays, sex and site had no main effects on relative liver mass (g/disk width; two-way ANOVA, *p* ≥ 0.28), although there was a site-sex interaction (*p* = 0.035). While mean relative liver mass was lowest in PCB-exposed males, post hoc testing found no differences among groups.

**Fig. 5:**
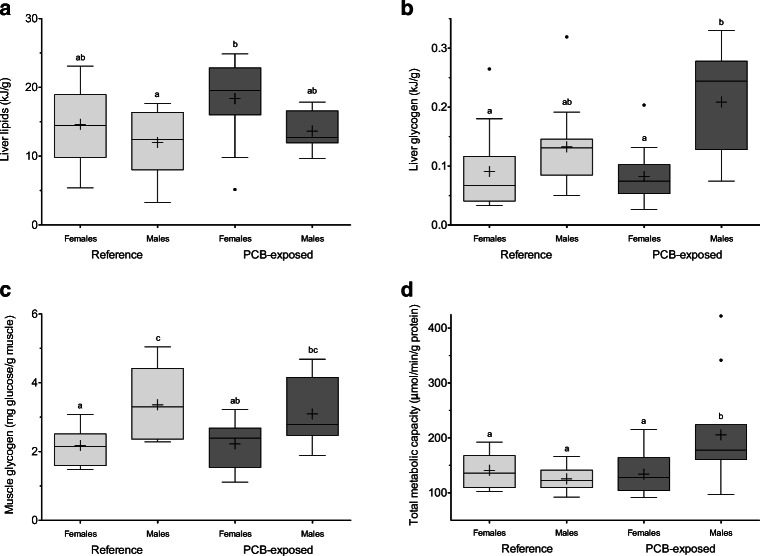
Comparisons of adult tissue quality for **a** liver lipids, **b** liver glycogen, **c** muscle glycogen, and **d** total metabolic capacity. Reference animals are shown in light gray bars and PCB-exposed in dark gray bars. Significant differences from two-way ANOVAs and Bonferroni post hoc correction for each parameter across groups are denoted with different letters. Whiskers of boxplots represent 1.5 times the interquartile difference and crosses within group means

Sex had a persistent effect on tissue quality measures. Despite the energetic burden of pregnancy, sex was the only significant factor for liver total energy content (two-way ANOVA, *p* = 0.019) and lipid content (*p* = 0.015) comparisons, with females generally having higher values than their male counterparts (Fig. 5a). In late-term PCB-exposed and reference females, lipid contributed 90 ± 5.7% and 87 ± 7.3% to total energy content, respectively, while contributions were 88 ± 3.7% and 82 ± 11% for males, respectively. Liver glycogen also showed a significant effect of sex (two-way ANOVA, *p* < 0.0001) as well as a significant interaction with site (*p* = 0.042). PCB-exposed males had the highest mean values and were significantly greater than both female groups in post hoc testing (*p* ≤ 0.003; Fig. 5b). Although reference males had higher mean values than females, they were not significantly different (*p* = 0.7). Liver protein content was the only energy substrate that did not differ among groups (*p* ≥ 0.13).

Muscle quality also differed between males and late-term pregnant females, with males following original predictions of having better measures than energetically taxed females. Sex and its interaction with site had a significant effect on muscle protein (two-way ANOVA, *p* < 0.0001 and *p* = 0.028, respectively). Reference males had the highest protein content of all groups (*p* ≤ 0.049), and males at both sites had higher content than their female counterparts (*p* ≤ 0.011). Muscle glycogen followed a similar pattern with sex having a significant effect (*p* = 0.0003). Reference males had significantly higher values than both female groups (*p* ≤ 0.029) with PCB-exposed males intermediate (Fig. 5c).

Site differences were more apparent for metabolic measures than tissue quality. For total metabolic capacity, site and its interaction with sex had significant effects on activity (two-way ANOVA, *p* = 0.033 and *p* = 0.004, respectively). PCB-exposed males had the highest activities of all groups (*p* ≤ 0.013; Fig. 5d), with no differences between reference males and females (*p* = 1.0). Among substrate groups, protein and carbohydrate metabolic capacity exhibited significant differences among sex-site groups, while ketone metabolic capacity was similar (*p* ≥ 0.07). For protein metabolic capacity, the interaction of sex and site was significant (*p* = 0.0006) as PCB-exposed males had higher activities than both reference groups (*p* ≤ 0.004) with PCB-exposed females intermediate. PCB-exposed males also exhibited a strong, negative association between liver protein content and protein metabolic capacity (*t*_9_ = − 4.6, *p* = 0.001, *r* = − 0.84; Supplemental Fig. 4D), while other groups showed only weak (reference females, *p* = 0.05) or no associations (*p* ≥ 0.3). Site and sex also had significant effects on carbohydrate metabolic capacity (two-way ANOVA, *p* = 0.0005 and *p* = 0.019, respectively), as PCB-exposed males and females had higher activities than reference females (*p* ≤ 0.016) with reference males intermediate.

## Discussion

Despite production bans nearly 50 years ago, PCBs continue to have adverse effects on wildlife health, highlighting the need to integrate toxicological assessments with management decisions. The large difference in mean PCB accumulation between sites cannot be discounted and is implicated in this study as a contributing factor for differences found between sites. While we documented clear variability in individual stingray PCB accumulation, which would decrease our power to detect differences, site was still identified as an important factor influencing physiological responses among groups. As such, potential PCB contaminant impacts on stingrays appear to not exert their effects equally between the sexes or across the life stages. This work extends our understanding of the implicated impacts of contaminant exposure on a model elasmobranch system using physiological biomarkers, and joins mounting evidence that environmental PCB exposure is likely not physiologically inert in elasmobranchs (Frantz 2014; Sawyna et al. 2017; Cullen et al. 2019) and may impose additional metabolic costs.

The variability in PCB congener structure and degree of chlorination results in multiple pathways by which PCBs can exert their effects with wide ranging biological consequences for organisms (Fischer et al. 1998; Jørgensen et al. 2006; Berninger and Tillitt 2019). Most well-studied is the induction of the aryl hydrocarbon receptor (AhR) cascade resulting in increased expression of the CYP1A detoxification enzyme (Nebert et al. 2000). However, PCBs can exert effects through non-AhR mechanisms such as acting as endocrine disruptors, particularly for important metabolic hormones such as thyroid hormone in teleost fishes (Brar et al. 2010). While we were unable to measure PCB contaminants in all study samples, the low levels of both dioxin-like PCBs and relative CYP1A expression suggest that any effect of PCB exposure is likely mediated through non-AhR pathways. Limited contaminant data also prevented us from relating apical endpoints measured in our study to contaminants accumulated due to low sample sizes across pregnancy stages. However, hepatic PCB concentrations similar to those measured in our study have induced metabolic impacts in fasting Arctic charr (*Salvelinus alpinus*; Vijayan et al. 2006) and are associated with growth and reproductive impairment in wild caught white sturgeon (*Acipenser transmontanus*; Feist et al. 2005). While sensitivity will differ across species (Eisner et al. 2016), PCB concentrations measured in our study were comparable with those known to elicit biological effects in other fishes.

### Effects on pregnant females

Pregnancy had a significant impact on many physiological parameters with negative outcomes occurring during the last stage when energetic demands on females are at their highest. As the major energy storage organ of elasmobranchs, the liver was more heavily taxed than overall relative body condition. The combination of both mass and lipid content decrease in the liver highlights the impact pregnancy has on liver quality and condition for late-term females.

Contrary to our initial expectations, PCB-exposed females had fairly large livers at the start of pregnancy (Fig. 1b) which were originally interpreted as females being in good condition, as HSI is an important factor used to assess health in fishes. This result is in contrast to other vertebrate studies where PCB exposure in the laboratory has been associated with lower liver mass loss compared to controls in exercised fish (van Ginneken et al. 2009) and higher liver mass gain in rats (Mehlman et al. 1974). As elasmobranch physiology differs from bony vertebrates in some key ways, interpretation of PCB effects on traditional biomarkers like HSI may be more complicated.

While pregnancy strains maternal resources, PCB exposure appeared to exacerbate loss, especially in muscle tissue (Fig. 1d). Quality in both muscle protein and glycogen stores significantly decreased in late-pregnancy for PCB-exposed females, whereas smaller but insignificant decreases were observed for reference females. Since muscle contributes largely to total body mass (~ 85%, Lyons, unpublished data), and represents a large energy cache for proteins and glycogen, any loss in quality could represent a substantial loss in overall body reserves for these substrates. Loss of muscle quality, exacerbated by PCB exposure, that impairs muscle function could have implications in swimming performance (Hopkins et al. 2003) and, thus, for predation vulnerability (Marentette et al. 2013), although this remains to be further investigated in elasmobranchs.

We hypothesized that PCB exposure would increase metabolic demands using enzyme activities as a proxy for cellular metabolism. Indeed, both carbohydrate and ketone metabolic enzymatic capacities were higher in PCB-exposed females. Higher activities may be an indicator that females are burning more energy for performing the “same task” (i.e., pregnancy) as reference females. Although protein metabolic capacity was not different across pregnancy or sites, PCB-exposed females consistently presented negative associations between liver protein content and protein metabolic activities at each stage. If we would have been able to track individual females across their entire pregnancy, we may have been able to determine the resulting outcome this may have on female tissue condition or her ability to provide nutrition to embryos by the end of pregnancy. In teleosts, effects of PCBs on metabolic enzymes and aerobic metabolism are mixed (van Ginneken et al. 2009; Wiseman and Vijayan 2011; Cannas et al. 2013). Considering the variables associated with this field study, further work is needed to delineate the potential role of individual PCB congeners and mixtures on elasmobranch hepatic metabolism through controlled exposures.

One downstream consequence of the additional energetic strain that may have been imposed by PCBs was an inability to maintain proper osmotic balance, particularly for plasma urea (Fig. 2). Urea is not only a foundation of elasmobranch osmoregulation, but is also energetically costly to produce; thus, maintaining concentrations are not only important for proper osmotic balance, but to reduce the loss of an energetically expensive molecule. The loss of urea in PCB-exposed females may indicate that urea retention ability and/or production was compromised directly or indirectly by PCB exposure. Reference females were found to have higher activities of key urea cycle enzymes, suggesting their capacity to produce urea was greater, but they also were able to better protect muscle protein stores (i.e., urea substrates) than PCB-exposed females. Considering that pregnancy represents a time of high energy demand and energy strain for females, the added cost of dealing with contaminant exposure may compromise osmoregulatory ability and/or efficiency. The negative correlation we found in late-term PCB-exposed females between mean litter mass and plasma urea concentrations may support this hypothesis. Our study suggests that urea may represent a sensitive indicator of physiological disruption.

### Effects on embryos

PCB burden was not quantified in embryos. The null hypothesis was that the embryonic PCB burden would be proportional to concentrations found in their mothers (Lyons and Lowe 2013). As such, site had several interacting effects along with development in embryos, with sex appearing as a factor in total metabolic capacity differences. Unlike adults that clearly have sex-related differences in physiological demands, male and female embryos were assumed to have similar energetic priorities and were exposed to similar environments within the womb. Therefore, it was significant that sex-related differences were even identified in utero suggesting exposure to maternally offloaded contaminants does not affect embryos equally. In this study, PCB-exposed male embryos were the only group to demonstrate significant increases in metabolic capacity (Fig. 4b), which could be a factor for why male embryos were not relatively heavier than females at the contaminated site as they were at the reference site (Lyons and Wynne-Edwards 2018). The extent to which these PCB-induced, sex-related differences in early development alter the lifetime metabolic trajectory of individuals and the downstream ramifications remain to be explored.

A potential consequence of early-developmental exposure to PCBs may be impaired embryonic growth through an inability to properly utilize maternally provided resources. Previously, we hypothesized that growth is more efficient in reference embryos since, despite a later start in development (reference females ovulated temporally later than PCB-exposed females), they were able to “catch up” to their PCB-exposed counterparts on a per mass basis within a few weeks (Lyons and Wynne-Edwards 2018). In the current study, we found embryo tissue quality measures to be higher in reference embryos, except for liver lipids (Fig. 3). As the main energy substrate utilized by elasmobranchs, lipids are likely an important energy substrate utilized by embryos to fuel their growth. The lower growth in PCB-exposed embryos suggests they were not using their lipids efficiently, appearing to shunt lipids towards liver storage rather than to somatic growth. In other vertebrates, exposure to PCB 153 is linked to lipid metabolic dysfunction by promoting lipid accumulation (Wahlang et al. 2013; Yadetie et al. 2014, 2017). Given the predominance of PCB 153 as one of the top accumulated congeners in stingray liver tissue, the adipogenic and lipid metabolic dysfunction effects of PCB 153 are implicated to occur in stingrays as well. Taken together, reference embryos appear to be more efficient at converting maternally supplemented energy into biomass than their mainland counterparts, suggesting PCB exposure in early development may contribute to energy utilization dysfunction. The production of less energetically efficient offspring could have survival implications after birth.

Despite the fact that round stingray embryos develop internally, enabling mothers the opportunity to manage osmoregulation, embryos appear to gain this capacity at a young age. While enzyme activities were quantified in optimal laboratory conditions, which may underestimate physiological limitations in vivo, we demonstrate key OUC enzymes are both present and capable of responding to developmental changes, suggesting they are functional. Furthermore, embryos appear to be able to maintain an osmotic boundary between themselves and surrounding histotroph, indicating they have some individual control of their internal osmotic environment. Using muscle as a whole-body proxy, urea muscle concentrations showed no change with time, despite the finding that urea histotroph in reference samples increased with development. However, embryos are not completely impermeable to urea uptake as embryo muscle urea was greater than in adults. Stingray embryos also appear capable of retaining and concentrating TMAO and this ability develops as they grow, similar to that documented in skate embryos developing outside their mother’s body in egg cases (Read 1968). In particular, despite the lower levels of histotroph TMAO in PCB-exposed samples, muscle tissue concentrations in these embryos were comparable to those in reference embryos, indicating they were able to still maintain comparable levels in the face of a limited external source (i.e., histotroph TMAO). However, muscle urea:TMAO ratios in stingray embryos were higher than those of oviparous skate embryos, which were capable of maintaining the desired 2:1 ratio (Steele et al. 2004), suggesting that the “environment” (i.e., uterine versus egg case) may have an influence on embryo osmoregulatory capacity.

Finally, since embryos must balance growth with storage, their energetic priorities are likely different than adults. This may result in energetic strategies changing across ontogeny. Embryos had differing composition of liver energy substrates compared to adults (Supplemental Information Fig. 7), with higher contributions of carbohydrates and proteins. In addition, embryos had lower metabolic capacities than adults (101 ± 19 versus 140 ± 68 μmoles product/min/gram protein), which could represent an energy-saving technique of the liver to allow more resources for extra-hepatic tissue growth or a reflection of the underdevelopment of this organ in utero. Thus, age should be considered in future studies when comparing energetic differences within or across species.

### Effects on adult males

Sex produced clear physiological differences between adult male and female stingrays. In teleosts, naturally higher metabolic rates in males are implicated in increasing PCB accumulation faster than in females (Madenjian 2020). In the present study, differences between tissue qualities reveal possible energetic priorities between males and females (Fig. 5). Despite females being at the height of their energetic demands, they had greater liver lipid content than males. This is noteworthy as lipid is the main energetic fuel stored by elasmobranchs as well as the most calorically dense. Males and females may have differing energetic priorities in what they store or how they chose to utilize energy. For instance, sex-related patterns in muscle contrasted those in liver where males had higher quality than females, suggesting females are willing to sacrifice muscle quality over liver quality.

Nevertheless, sex appears to be an important factor in how response to toxicant stressors manifests itself in individuals, which has been identified in other vertebrates across life stages (Fernie et al. 2003; van Esterik et al. 2015; Espín-Pérez et al. 2019). In teleosts, sex-related differences in liver biomarker response to PCB exposure have been observed in adults (Vega-López et al. 2007; Li et al. 2019), suggesting that sex must be considered when determining potential contaminant impacts to individuals or populations. Here, we indicate that PCB exposure may modulate sex-specific differences in elasmobranchs as well. For liver glycogen, males had higher concentrations than females; however, PCB-exposed males had particularly high concentrations. A previous work has suggested potential carbohydrate metabolic dysfunction, especially in response to acute stress (Lyons and Wynne-Edwards 2019b), which may contribute to the overaccumulation of this substrate in PCB-exposed males. Similarly, although non-sex-related, hepatic metabolic disturbances were found in rainbow trout exposed to Aroclor 1254 (PCB industrial mixture) (Wiseman and Vijayan 2011), suggesting carbohydrate processing may be a target of PCB exposure. In addition, PCB-exposed males had the highest metabolic capacity of all groups, as a result of having the highest mean protein and carbohydrate metabolic capacities.

Using enzyme activities as a proxy for overall metabolism, we may interpret PCB-exposed males as having the highest capacity to burn energy. This was unexpected considering late-term females are under strenuous energetic demands and were predicted to have higher enzyme activities than males. However, the patterns found in reference animals were closer to our predictions as metabolic capacity in reference males was no different in females from either group. The elevated potential for energy generation in PCB-exposed males, during a period when they are least reproductively active, may indicate additional energetic burdens not faced by reference males, such as those imposed by dealing with contaminant stressors. Indeed, previous works have demonstrated elevated CYP1A content and EROD activity in PCB-exposed males compared to their reference counterparts (Lyons et al. 2014). The downstream consequences of higher metabolic demands potentially without accompanying better tissue quality remain to be fully examined, but may be indicative of broader physiological outcomes (such as growth rate, reproductive success) hampered in PCB-exposed males compared to reference males.

### Environmental implications

Concentrations of PCB contaminants measured in round stingrays and the associated, sublethal physiological impairments listed here suggest that environmental exposure to these animals is not negligible. Further connections are needed to link elasmobranch ecology with toxicology to better understand risk of exposure, as has been performed in teleost fishes (Teesdale et al. 2015; Wolfe and Lowe 2015), and to determine variation in exposure across an animal’s lifetime. Understanding how animals utilize particular environments may inform remediation efforts along with continued monitoring. Assessments of sediment chemistry and toxicity from many locations across southern California’s coastline (1–200 m depth), estuaries, and embayments take place every 2 to 5 years through the Southern California Bight Regional Monitoring Program. Toxicity is determined based on adult amphipod (*Eohaustorius estuarius*) and embryo mussel (*Mytilus galloprovincialis*) survival tests that constitutes one of three prongs used to evaluate sediment quality and status. From the most recent report, embayments and brackish estuaries were more impacted than the exposed coastline, regardless of depth tested (Parks et al. 2020). The former two correspond to areas that stingrays use as part of their natural life cycle, and may represent sites of significant contaminant exposure. More work is needed to determine how well these invertebrate toxicity tests, a well utilized system to rapidly assess sediment toxicity, correlate or predict sublethal effects measured in higher level predators such as the round stingray or other teleost fishes that utilize these environments. Utilizing one or two model species for environmental evaluation may not capture the full impact of contamination on an ecosystem considering that effects may vary across species, life stages, and sexes. Thus, inclusion of non-traditional species is needed in order to holistically determine the extent to which contaminants may be influencing ecosystem health.

## Conclusions

Legacy PCB contamination continues to be implicated for adverse outcomes on round stingray homeostasis, manifested as higher metabolic burden, lower energy reserves, and production of lower quality embryos. The association of PCBs with sublethal endpoints in round stingray, a mesopredator occupying lower trophic levels, suggests other elasmobranchs may be vulnerable to organochlorine toxicity, although they remain understudied. In particular, sex appears to be a factor for how animals experienced effects implicated by PCB exposure, with males being more sensitive at both ends of ontogeny (i.e., embryos and adults). Future work should continue the investigations into potential sublethal impacts of contaminant on elasmobranch physiology, including extending to species with different life history strategies, to determine population-level consequences (if any) and their implications for species management strategies and ecosystem health.

## Supplementary information

ESM 1 (DOCX 2127 kb)

## Data Availability

The datasets used and/or analyzed during the current study are available from the corresponding author on reasonable request.

## Footnotes

1 https://oehha.ca.gov/advisories/santa-monica-beach-south-santa-monica-pier-seal-beach-pier
