# Supplementary material for: Sublethal, sex-specific, osmotic, and metabolic impairments in embryonic and adult round stingrays from a location exposed to environmental contamination in southern California, USA
Source: Environ Sci Pollut Res Int. 2021 Jan 28;28(22):27493–510. doi: 10.1007/s11356-021-12546-0 (PMC8164579; doi:10.1007/s11356-021-12546-0)
Supplement: Supplementary file 1 — (DOCX 2127 kb) [file 11356_2021_12546_MOESM1_ESM.docx]

**Supplemental Information**

Title: Sublethal, sex-specific, osmotic and metabolic impairments in embryonic and adult Round Stingrays from a location exposed to environmental contamination in southern California, USA

Authors: Kady Lyons^1,2*^, Katherine E. Wynee-Edwards^3^

1. University of Calgary, Department of Biological Sciences, 2500 University Dr. NW, Calgary, AB, T2N 1N4, Canada; [kady.lyons@sbcglobal.net](mailto:kady.lyons@sbcglobal.net)
2. Georgia Aquarium, 225 Baker St NW,
3. Faculty of Veterinary Medicine, University of Calgary, 3280 Hospital Dr NW, Calgary, AB, T2N 4Z6, Canada

Journal: Environmental Science and Pollution Research

**Capture stress experiment details**

Details on the capture stress experiment can be found in: Kady Lyons & Katherine E. Wynne-Edwards (2019) Legacy environmental polychlorinated biphenyl contamination attenuates the acute stress response in a cartilaginous fish, the Round Stingray, *Stress*, 22:3, 395-402, DOI: 10.1080/10253890.2019.1570125

Briefly, stingrays were captured at two locations: Seal Beach National Wildlife Refuge, mainland southern California (PCB-exposed site), and Catalina Harbor, Santa Catalina Island (reference site). All animals were captured one at a time via hook and line from the shoreline. For animals in our “baseline” group, once an animal was hooked, it was reeled in immediately, euthanized and tissues were immediately sampled and placed on dry ice in the field. Time from from hooking to euthanasia was under 2 mins and represented the closest possible baseline values we could obtain for a field study. For our “stressed” group, once animals were hooked they were allowed time to fight the line by reeling them in slowly (~5 mins), after which they were placed in a bucket with ambient seawater and a bubbler for 15 mins before euthanasia and tissue sampling. All activities were approved by the University of Calgary under protocol AC#14-0016.

Table 1. Mean ± standard deviation activities (µmoles/min/g) of adult hepatic enzyme activities by site and sex for late-term pregnant females and reproductively inactive adult males. Different letters represent significant differences among sex-site groups for each enzyme via two-way ANOVA followed by posthoc tests with Bonferroni correction.

|  | Reference Females  (n = 8) | Reference  Males  (n =8) | Exposed  Females  (n =10) | Exposed  Males  (n = 11) |
| --- | --- | --- | --- | --- |
| Alanine aminotransferase | 78.9 ± 18.5^ab^ | 60.8 ± 11.9^a^ | 65.6 ± 22.6^a^ | 105.7 ± 55.76^b^ |
| Aspartate aminotransferase | 36.8 ± 11.4^a^ | 32.7 ± 6.60^a^ | 37.0 ± 11.0^a^ | 57.19 ± 26.62^b^ |
| Hexokinase | 4.59± 1.39^a^ | 4.08 ± 1.43^a^ | 4.31 ± 1.18^a^ | 5.48 ± 2.60^a^ |
| Glutamate dehydrogenase | 9.45 ± 3.90^a^ | 9.59 ± 2.95^a^ | 6.22 ± 1.89^a^ | 14.24 ± 6.03^b^ |
| Lactate dehydrogenase | 5.96 ± 1.47^ab^ | 5.09 ± 0.994^a^ | 7.55 ± 2.29^bc^ | 8.89 ± 3.27^c^ |
| Phosphoenolpyruvate carboxykinase | 0.204 ± 0.235^a^ | 0.555± 0.653^ab^ | 0.307 ± 0.412^ab^ | 0.817 ± 0.611^b^ |
| Pyruvate kinase | 4.91 ± 2.34^a^ | 12.4 ± 4.37^b^ | 12.6 ± 5.31^b^ | 13.0 ± 6.29^b^ |
| 3-hydroxybutyrate dehydrogenase | 0.278 ± 0.141^a^ | 0.360 ± 0.169^a^ | 0.481 ± 0.259^a^ | 0.362 ± 0.211^a^ |

Table 2. Mean ± standard deviation activities (µmoles/min/g) of embryo hepatic enzyme activities by site and sex. Enzyme activities were analyzed from one embryo of each litter starting from clasper day 0 (total mass ~3g) from each site, where sufficient tissue could be extracted. Note that development and sex had significant effects on some enzyme activities as indicated below.

|  | Reference Females  (n = 11) | Reference  Males  (n = 10) | PCB-Exposed  Females  (n = 8) | PCB-Exposed  Males  (n = 11) |
| --- | --- | --- | --- | --- |
| Alanine aminotransferase* | 31.99 ± 11.59 | 36.36 ± 12.83 | 25.58 ± 10.20^b^ | 36.30 ± 11.97^b^ |
| Aspartate aminotransferase* | 36.12 ± 17.96^a^ | 27.20 ± 15.94^a^ | 24.89 ± 18.31 | 24.89 ± 11.76 |
| Hexokinase | 6.16 ± 0.59 | 5.90 ± 0.38 | 5.96 ± 0.66 | 6.07 ± 0.42 |
| Glutamate dehydrogenase | 9.36 ± 3.53^a^ | 9.41 ± 4.50 ^a^ | 6.59 ± 3.13 ^a^ | 7.75 ± 1.89 ^a^ |
| Lactate dehydrogenase | 14.43 ± 9.32 | 12.32 ± 2.37 | 10.53 ± 2.64 | 14.16 ± 4.84 |
| Phosphoenolpyruvate carboxykinase | 0.93 ± 1.48 | 1.83 ± 2.09 | 0.56 ± 0.24 | 2.08 ± 2.96 |
| Pyruvate kinase | 11.39 ± 3.48 | 10.19 ± 2.62 | 10.18 ± 2.99 | 11.97 ± 3.31 |
| 3-hydroxybutyrate dehydrogenase† | 0.08 ± 0.06 | 0.07 ± 0.05^c^ | 0.11 ± 0.07 | - 1. ± 0.04^b^ |

1. Embryos showed decreases in activity over development
2. Embryos showed increases in activity over development
3. Embryos with weak increases (p = 0.063)

* Enzymes with different responses between sites

† Enzymes with different responses by sex


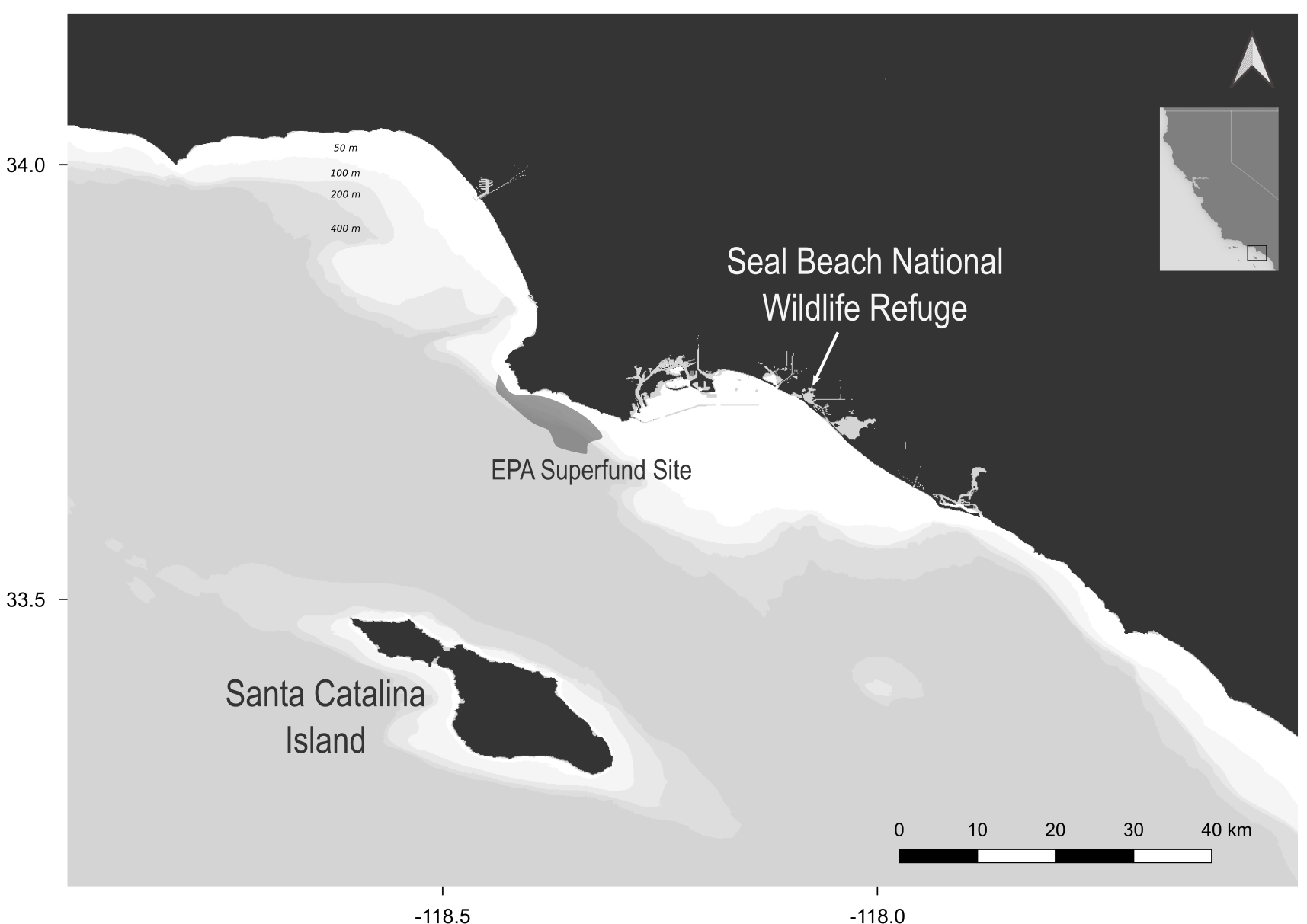


**Figure 1.** Stingrays were sampled from two areas in southern California. Seal Beach Ntional Wildlife Refuge (mainland California) represented our PCB-exposed site and Santa Catalina Island, located ~35 km offshore, represented our reference site. Area representing the Palos Verdes US Environmental Protection Agency superfund site is shown in light grey. Bathymetry is indicated, with darker colors corresponding to deeper depths.


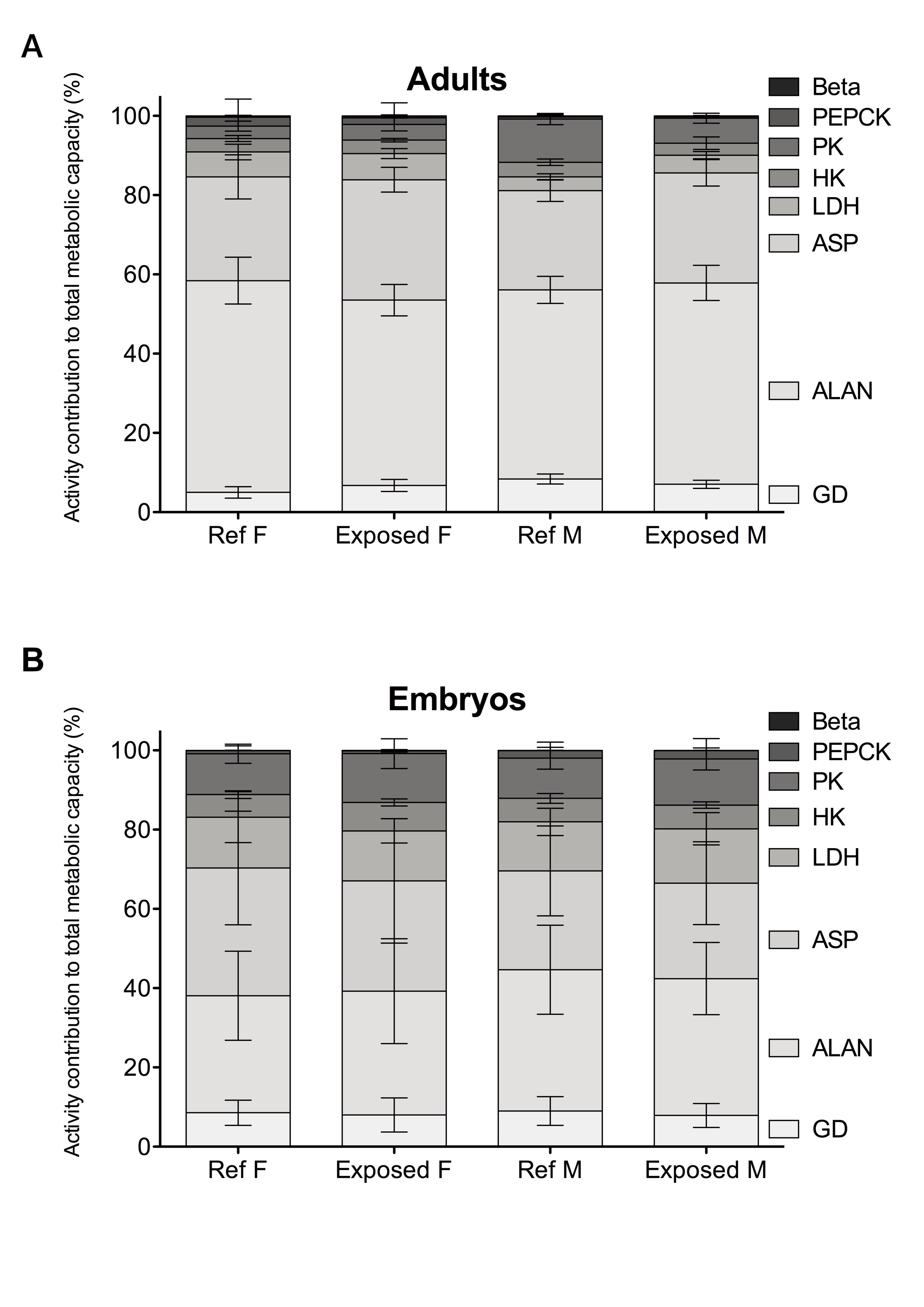


**Figure 2.** Relative enzyme activities as they contribute to total activity for adults (A) and embryos (B) separated by sex-site groups. Different enzymes are shown in different shades of grey. GD = Glutamate dehydrogenase, ALAN = Alanine aminotransferase, ASP = Aspartate aminotransferase, LDH = Lactate dehydrogenase, HK = Hexokinase, PK = Pyruvate kinase, PEPCK = Phosphoenolpyruvate carboxykinase, Beta = 3-hydroxybutyrate dehydrogenase.


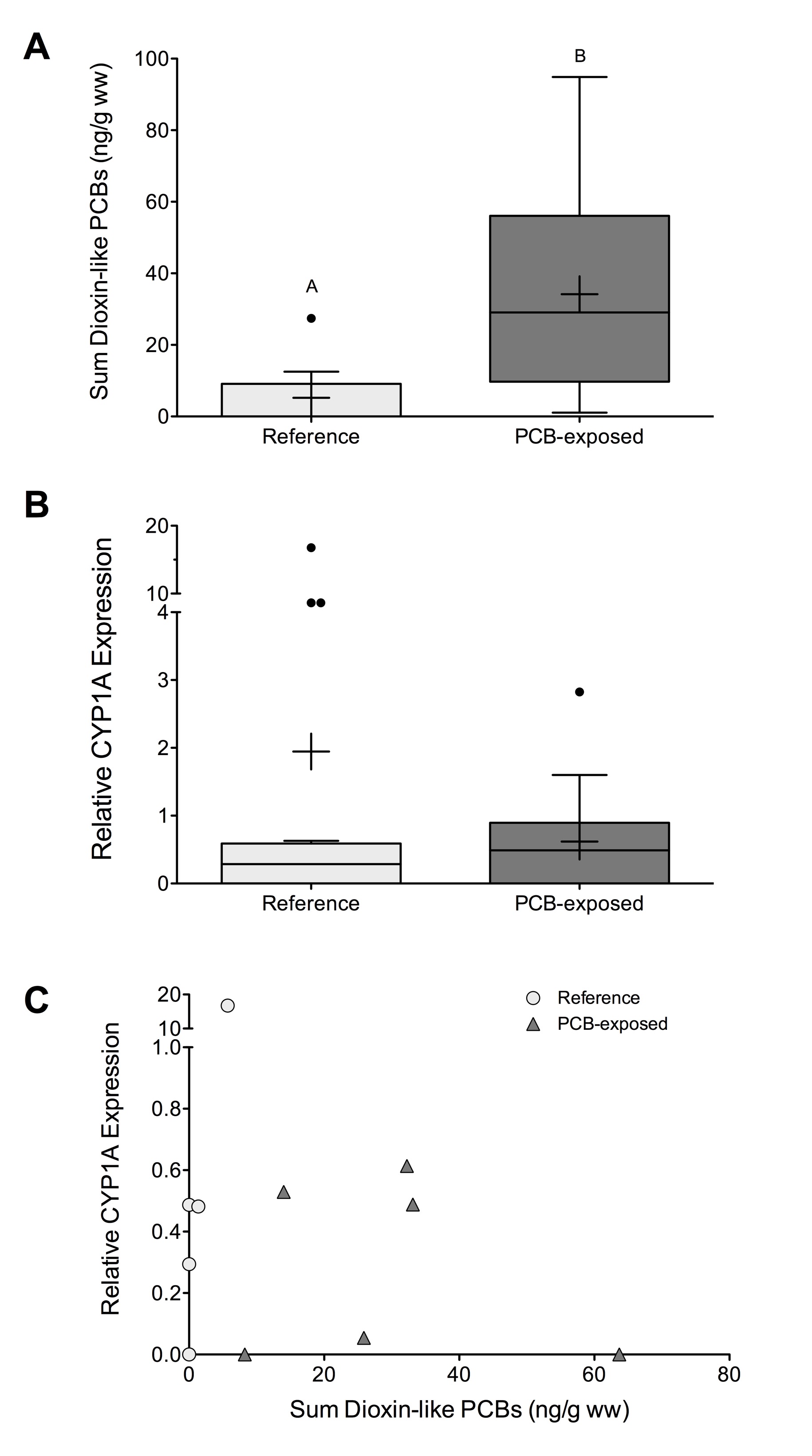


**Figure 3.** Summed concentrations of dioxin-like (i.e. planar) PCB contaminants were significantly higher (Mann-Whitney U test, p = 0.003) in PCB-exposed females compared to reference female livers (A). CYP1A expression was assessed via Western Blot and expression was quantified as spectral absorbance under the peak relative to β-actin expression for each sample of mid to late-term female livers from each site (B). Summed dioxin-like PCBs were compared to the expression of relative CYP1A for PCB-exposed (triangles) and reference (circles) females.


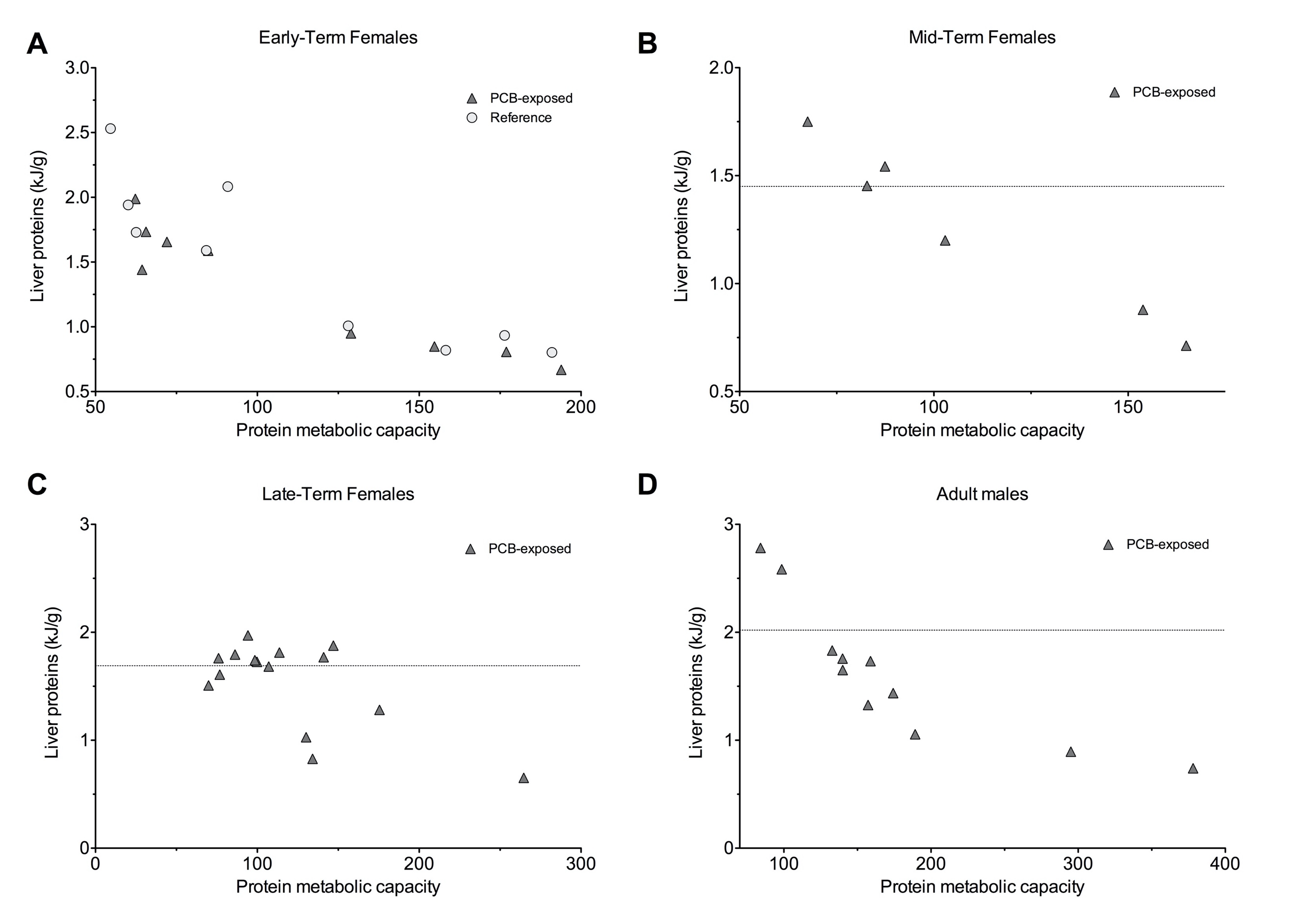


**Figure 4.** Significant correlations between protein metabolic capacity and liver protein content for females during early (A), mid (B) and late (C) pregnancy and adult males are shown. PCB-exposed animals are shown in triangles and reference animals in circles. For panels B and C, dashed horizontal line represents mean liver protein content in reference females for the corresponding stage. Similarly, in panel D dashed line represents mean liver protein content in reference males.


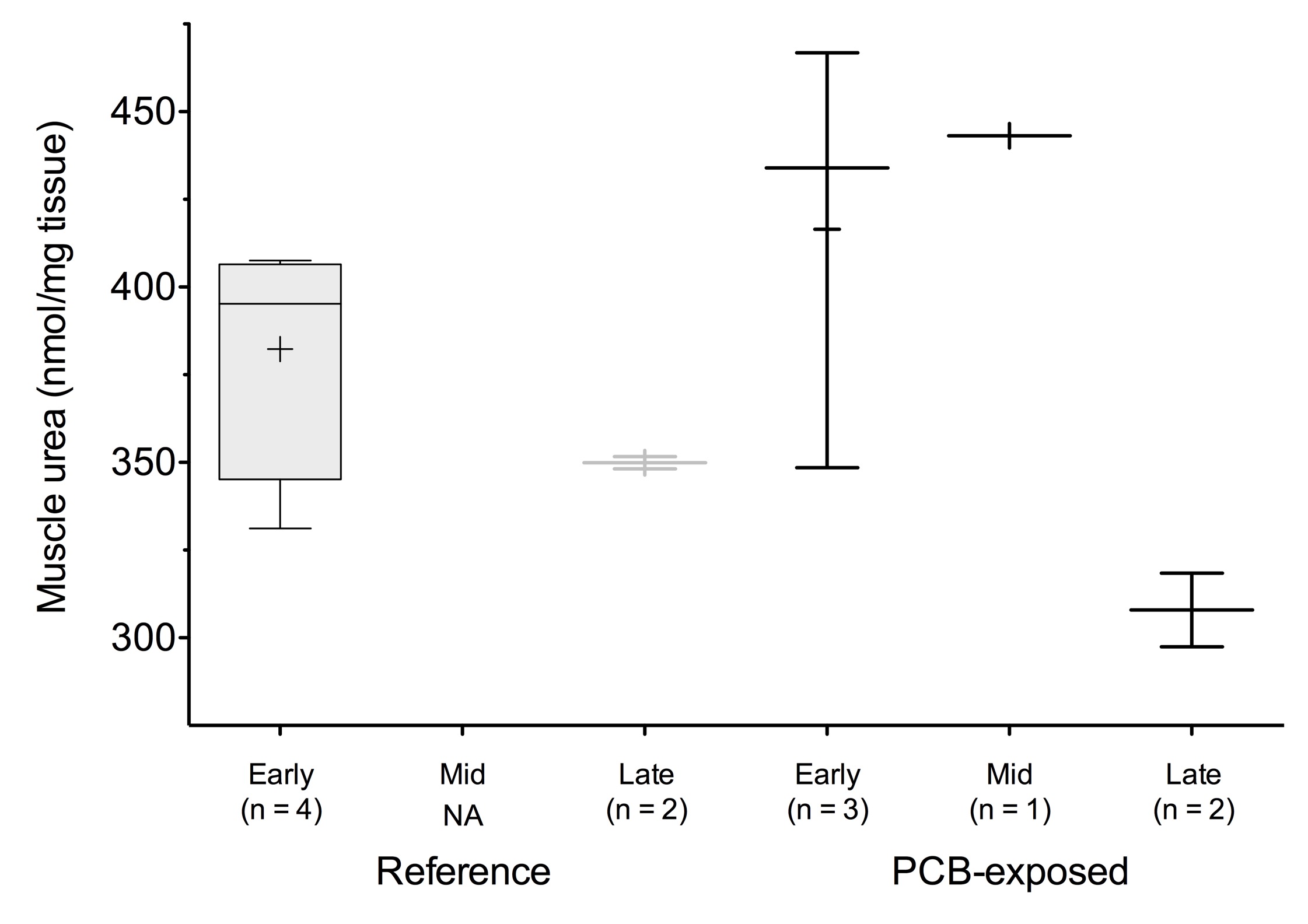


**Figure 5.** Muscle urea from a subset of pregnant females from the reference (light grey bars) and PCB-exposed (black bars) sites across pregnancy. Sample numbers for each category are given below each stage.


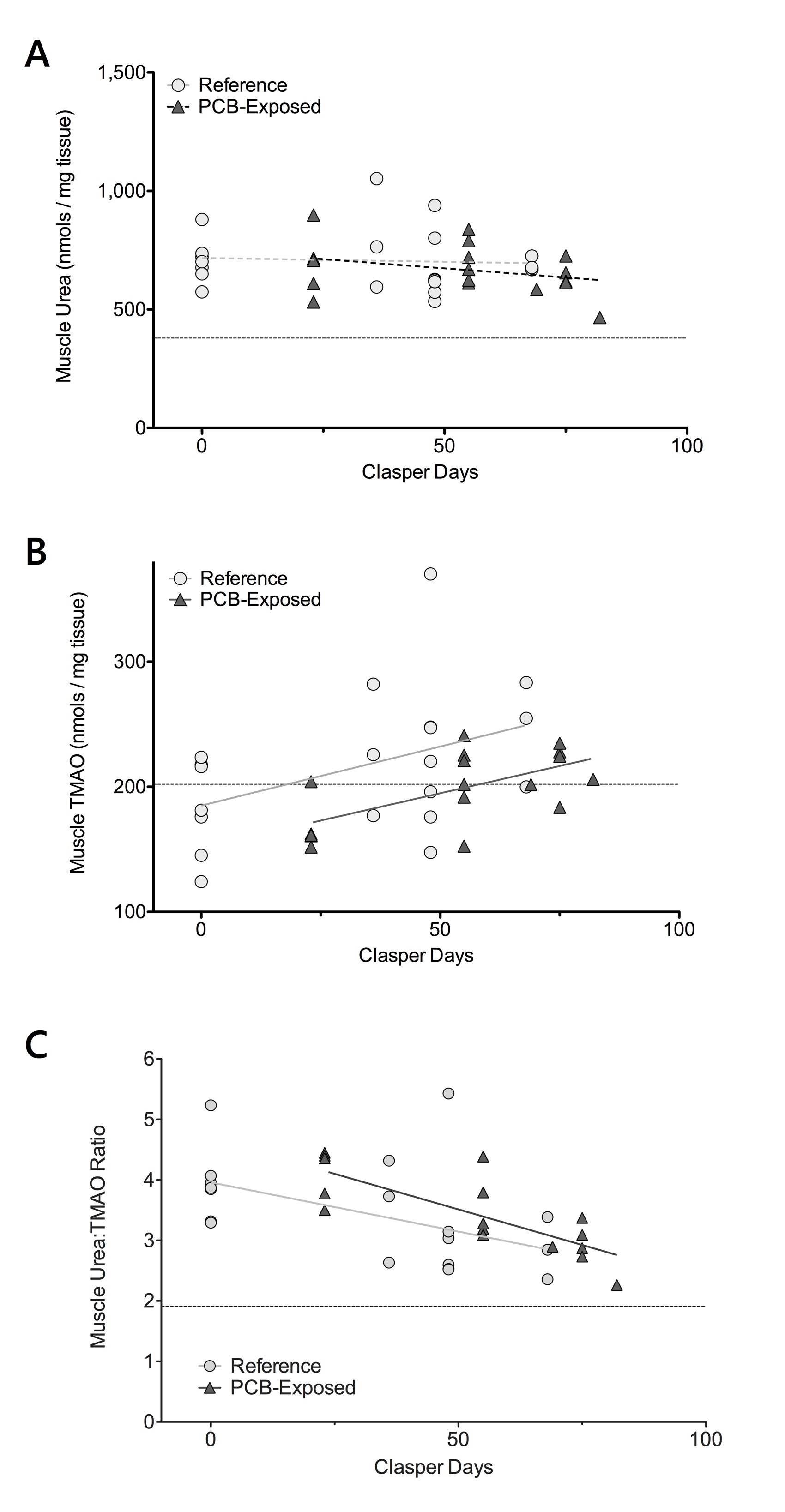


**Figure 6.** Muscle urea (A) and muscle TMAO (B) was measured in one embryo from each litter over development from reference (circles) and PCB-exposed (triangles) samples, with respect to mean adult muscle concentrations (horizontal dashed line). (C) Muscle Urea:TMAO ratios significantly decreased with embryo development, although they were higher than the optimal 2:1 ratio in adults (Yancey and Somero, 1980), which was replicated in this study (dashed line for mean adult female ratio). Significant relationships (p < 0.05) are shown in solid lines and insignificant relationships in dashed lines.


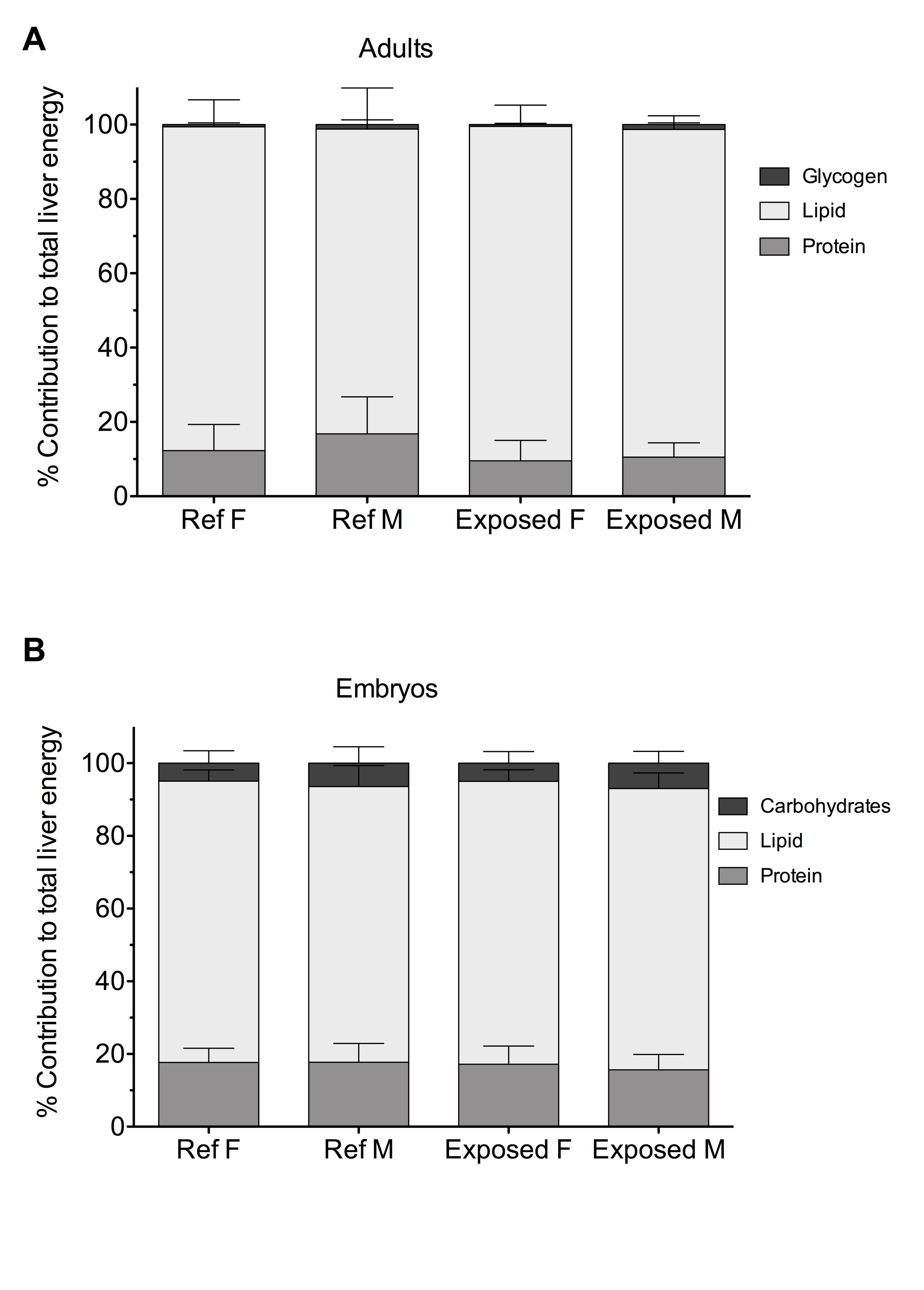


**Figure 7.** Mean and standard deviation of liver energy contributions from each substrate group for adult (A) and embryo (B) males and females. Since our imposed acute stressor influenced adult glucose only glycogen energy contributions are shown for adults, whereas carbohydrates encompass both glucose and glycogen stores in embryos.
